# Acute kidney injury due to anti-tuberculosis drugs: a five-year experience in an aging population

**DOI:** 10.1186/1471-2334-14-23

**Published:** 2014-01-13

**Authors:** Chia-Hao Chang, Yen-Fu Chen, Vin-Cent Wu, Chin-Chung Shu, Chih-Hsin Lee, Jann-Yuan Wang, Li-Na Lee, Chong-Jen Yu

**Affiliations:** 1 Department of Internal Medicine, National Taiwan University Hospital, Hsin-Chu Branch, Taiwan; 2 Department of Internal Medicine, National Taiwan University Hospital, Yunlin Branch, Taiwan; 3 Department of Internal Medicine, National Taiwan University Hospital, Taipei, Taiwan; 4 Department of Traumatology, National Taiwan University, Hospital, Taipei, Taiwan; 5 Department of Internal Medicine, Buddhist Tzu Chi General, Hospital-Taipei Branch, New Taipei, Taiwan; 6 Department of Laboratory Medicine, National Taiwan University Hospital, Taipei, Taiwan

**Keywords:** Acute kidney injury, Anti-tuberculosis drug, Fever, Rash, Rifampin

## Abstract

**Background:**

Patients on anti-tuberculosis treatment may develop acute kidney injury (AKI), but little is known about the renal outcome and prognostic factors, especially in an aging population. This study aimed to calculate the incidence of AKI due to anti-TB drugs and analyze the outcomes and predictors of renal recovery.

**Methods:**

From 2006 to 2010, patients on anti-TB treatment were identified and their medical records reviewed. Acute kidney injury was defined according to the criteria established by the AKI Network, while renal recovery was defined as a return of serum creatinine to baseline. Predictors of renal recovery were identified by Cox regression analysis.

**Results:**

Ninety-nine out of 1394 (7.1%) patients on anti-TB treatment had AKI. Their median age was 68 years and there was male predominance. Sixty (61%) developed AKI within two months of anti-TB treatment, including 11 (11%) with a prior history of rifampin exposure. Thirty (30%) had co-morbid chronic kidney disease or end-stage renal disease. The median time of renal recovery was 39.6 days (range, 1–180 days). Factors predicting renal recovery were the presence of fever, rash, and gastro-intestinal disturbance at the onset of AKI. Sixty-two of the 71 (87%) patients who recovered from AKI had successful re-introduction or continuation of rifampin.

**Conclusions:**

Renal function impairment is not a rare complication during anti-TB treatment in an elderly population. The presence of fever and rash may be associated with renal recovery. Rifampin can still be used in most patients who recover from AKI.

## Background

Tuberculosis (TB) is a global disease affecting one-third of the world’s population [1]. In 2011, around 60% of new TB cases worldwide were in Asia [2]. In Taiwan, the incidence of TB is around 57 cases per 100,000 population [3]. Prompt anti-tuberculosis treatment remains the most important and effective intervention for controlling spread, but adverse events from first-line anti-TB drugs are not uncommon. Acute kidney injury (AKI) is a rare and severe complication that can interrupt treatment and cause permanent kidney damage [4]. Although isoniazid (INH) and ethambutol (EMB) have been associated with AKI [5,6], rifampin (RIF) is the most common anti-TB drug responsible for AKI identified by most studies [7-10].

Reviewing literature of rifampin-induced AKI [4,11-13], the mean age of reported cases is around 40–45 years and the recovery rate ranges from 83% to 96%. However, the incidence to rifampin-induced AKI is uncertain because the definitions of AKI used in previous studies vary. The largest study disclosed that 60 out of 120,132 (0.05%) patients developed AKI [11]. Because the elderly are more vulnerable to drug-induced AKI and usually have poor renal recovery [14,15], AKI during anti-TB treatment may be more common and serious in Taiwan, where more than 50% of TB patients are older than 65 years. Furthermore, prognostic factors have never been investigated.

The present study was conducted to investigate the incidence of AKI during anti-TB treatment in Taiwan and to determine outcomes and predictive factors for renal recovery.

## Methods

### Study population

This retrospective study was conducted at the National Taiwan University Hospital. The hospital’s Research Ethics Committee approved the study protocol. Patients were included if they met the following criteria: (1) age ≥18 years; (2) clinical diagnosis or suspicion of TB; (3) under rifampin-containing anti-TB treatment; and (4) had onset of AKI during anti-TB treatment. Acute kidney injury (AKI) was defined according to the criteria established by the Acute Kidney Injury Network (AKIN) and was classified into three stages (Stages 1 to 3) based on serial changes in serum creatinine level. Stage 1 was defined as an increase in serum creatinine ≥26.52 μmol/L or by 1.5-fold but less than twice the baseline level. Stage 2 was defined as a two-fold increase but less than three-fold increase from baseline, while Stage 3 was defined as a three-fold increase from the baseline level [16].

Based on the *Taiwan Guidelines for TB Diagnosis and Treatment*[17], all TB patients received a standard anti-TB treatment of daily INH, RIF, EMB, and pyrazinamide (PZA) for the first two months, and daily INH and RIF for the next four months. For patients with an estimated creatinine clearance of < 30 ml/minute, the frequencies of EMB and PZA were changed to once every two days with the unit dose unchanged. The regimen was modified by the primary care physician if necessary, e.g. when there were adverse drug effects.

Patients were excluded if they: 1) had shock or urinary tract infection; 2) were under potentially nephrotoxic drugs other than rifampin at the onset of AKI; 3) had other conditions possibly resulting in AKI, such as hypercalcemia and nephrotic syndrome; 4) had end-stage renal disease and was under renal replacement therapy; and 5) had non-tuberculous mycobacteria infection.

### Data collection

Demographic data, including sex, age, smoking status, excessive alcohol consumption (defined according to a single-question alcohol screening test) [18], co-morbidities, results of sputum acid-fast bacilli (AFB) smear and mycobacterial culture, anti-TB regimen, laboratory results, onset, and management of AKI, were collected. Chronic kidney disease was defined according to the National Kidney Foundation Kidney Disease Outcomes Quality Initiative (K/DOQI) clinical practice guidelines, with an estimated glomerular filtration rate (eGFR) of < 60 mL/min/1.73 m^2^ for three months or more [19]. Baseline laboratory tests included a hemogram, renal function (blood urea nitrogen [BUN], creatinine) tests, and levels of liver enzymes (aspartate aminotransferase and alanine aminotransferase), total bilirubin, albumin, and uric acid.

The patients were classified into two groups based on hemoglobin < or ≥100 g/L, leukocyte > or ≤10×10^9^/L, eosinophil count > or ≤0.5×10^9^/L, and platelet < or ≥100×10^9^/L. Hepatitis was defined as increased serum alanine aminotransferase >3 times the upper limit of normal (ULN) in symptomatic patients, or >5 times the ULN in asymptomatic patients, or serum total bilirubin >51.3 μmol/L [17,20]. Hypoalbuminemia was defined as albumin < 35 g/L, hematuria as urine red blood cell >5 per high-power field (HPF), sterile leukocyturia as urine leukocyte >5 per HPF with negative urine bacterial culture, and proteinuria as urine protein >30 mg/dL. Based on the Taiwan treatment guidelines for TB, laboratory tests were repeated every two weeks in the first two months and every eight weeks thereafter [17], or when the primary care physician deemed it necessary. The time to AKI was defined as the interval between the start of anti-TB treatment and the onset of AKI.

Renal recovery was defined as a return of serum creatinine to baseline and the absence of AKI features. Time to recovery was defined as the interval between the onset of AKI and renal recovery. If renal recovery was not achieved after 180 days from the onset of AKI, the AKI was considered “unrecovered”.

### Statistical analysis

All data were expressed as either mean ± standard deviation or median [inter-quartile range]. Inter-group difference was compared using the *t*-test or Mann–Whitney *U*-test for continuous variables based on their normality, and the *chi*-square test or Fisher’s exact test for categorical variables, as appropriate. Time to renal recovery for each variable was compared using the Kaplan-Meier method with log-rank test. All variables with a *p* value ≤0.1 in univariate analysis were entered into a multivariate Cox proportional hazards regression analysis to compute the adjusted hazard ratios (HR) and 95% confidence intervals (CI). Variance inflation factor (VIF) was used to quantify the severity of multi-collinearity. All analyses were conducted using the Statistical Package for the Social Sciences (SPSS, v. 18.0; SPSS Inc, Chicago, IL). Statistical significance was set at *p* < 0.05. Sensitivity analysis was performed in the sub-population without CKD, since it was difficult to differentiate an “acute-on-chronic” disease from progression of CKD [16].

## Results

### Patient characteristics

**Figure 1:**
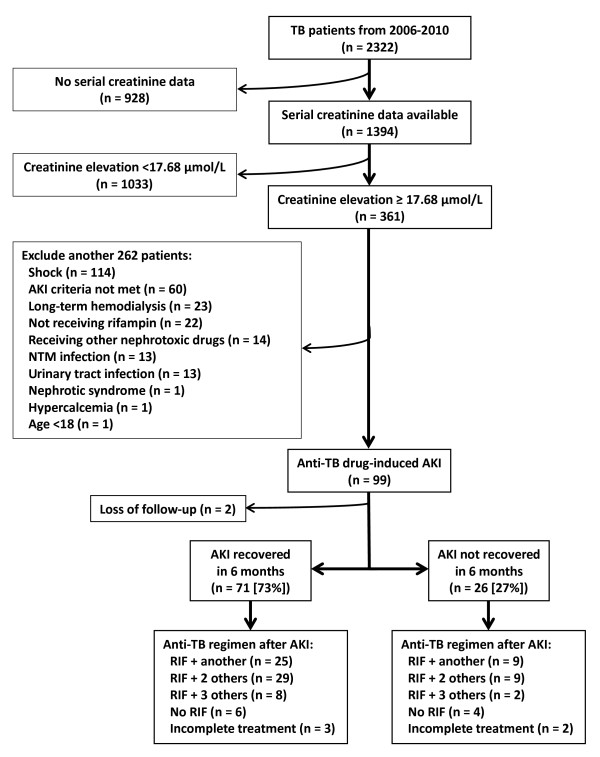
**Selection and disposition of study subjects.** AKI, acute kidney injury; NTM, non-tuberculous mycobacteria; TB, tuberculosis; RIF, rifampin**.**

From 2006 to 2010, 2322 TB patients were identified, including 1394 with serum creatinine data before and after the start of anti-TB treatment. Of the 361 patients with increased serum creatinine level ≥17.68 μmol/L, 262 were excluded (Figure 1) and only 99 (7.1%) were included for further analysis. In terms of severity, 83 (84%) patients were in AKIN Stage 1, 10 (10%) in Stage 2, and 6 (6%) in Stage 3. The patients’ median age was 68 years (IQR, 56–76 years), and there was a male predominance (71%). The diagnosis of TB was culture-confirmed in 80%. Of the 99 AKI patients, 16% had regular alcohol intake and 49% were smokers (Table 1). The most common underlying co-morbidities were pre-existing chronic kidney disease, diabetes mellitus, and malignancy.

**Table 1:** Patient characteristics based on recovery status of acute kidney injury (AKI)

**Variable**	**Overall (n = 99)**	**AKI-recovered (n = 71)**	**AKI-unrecovered (n = 26)**	**Loss of follow-up (n = 2)**
Male	70 (71)	51 (72)	18 (69)	1 (50)
Age ≥65	59 (60)	42 (59)	16 (62)	1 (50)
Smoking	48 (49)	37 (52)	10 (38)	1 (50)
Alcoholism	16 (16)	13 (18)	2 (8)	1 (50)
Malnutrition	39 (39)	24 (34)	14 (54)	1 (50)
Old TB history	3 (3)	2 (3)	1 (4)	0 (0)
Re-treatment of TB*	11 (11)	9 (13)	2 (8)	0 (0)
Co-morbidity				
CKD	30 (30)	21 (30)	9 (35)	0 (0)
DM	25 (25)	15 (21)	10 (38)	0 (0)
Malignancy	25 (25)	19 (27)	5 (19)	1 (50)
Gout	15 (15)	10 (14)	5 (19)	0 (0)
Autoimmune disease	6 (6)	4 (6)	2 (8)	0 (0)
HIV	2 (2)	2 (3)	0 (0)	0 (0)
Sputum mycobacterial study				
AFB-positive	29 (29)	22 (31)	6 (23)	1 (50)
Culture-positive	79 (80)	56 (79)	21 (81)	2 (100)
Presentations of AKI				
Rash	21 (21)	18 (25)	3 (12)	0 (0)
Gastro-intestinal upset	17 (17)	14 (20)	3 (12)	0 (0)
Fever	6 (6)	5 (7)	1 (3.8)	0 (0)
Arthralgia	4 (4)	4 (6)	0 (0)	0 (0)
AKI stage				
Stage 1	83 (84)	63 (89)	19 (73)	1 (50)
Stage 2	10 (10)	6 (8)	3 (12)	1 (50)
Stage 3	6 (6)	2 (6)	4 (15)	0 (0)
Onset of AKI after ATT (days)	44 [20–102]	40 [15–104]	50 [27–91]	73 [44–102]
Management after AKI				
Hold rifampin	34 (34)	22 (31)	10 (38)	2 (100)
Hold pyrazinamide^#^	35 (51)	24 (28)	11 (42)	0 (0)
Re-challenge rifampin	21 (21)	14 (20)	7 (27)	NA

*Abbreviations:AFB* acid-fast bacilli smear, *AKI* acute kidney injury, *ATT* anti-TB treatment, *TB* tuberculosis.

Note: Data are either number (%) or median [inter-quartile range]. There was no statistically significant difference between the AKI-recovered and -unrecovered groups.

*Re-treatment meant that AKI recurred after re-exposure to rifampin.

^#^Only 69 patients received pyrazinamide-containing anti-TB regimen at the onset of AKI.

### Onset of AKI

**Figure 2:**
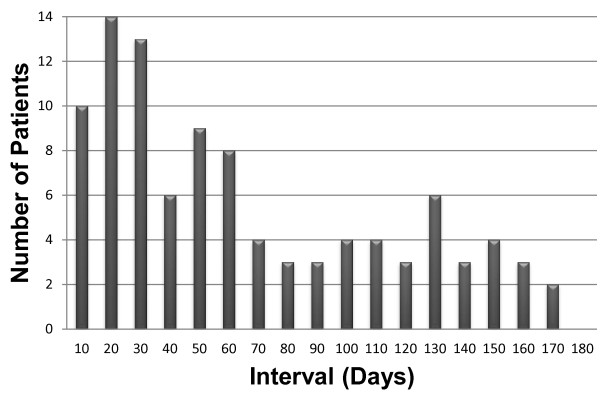
Interval between the start of anti-tuberculous treatment and onset of acute kidney injury.

Within six months after anti-TB treatment, there was a continuous probability of developing AKI (Figure 2). The median interval in all of the study subjects between the start of anti-TB treatment and the onset of AKI was 44 days [IQR, 20–102]. Moreover, 61% of AKI episodes happened in the first two months of treatment. In all patients taking rifampin at the onset of AKI, 97 (98%) were also taking isoniazid, 82 (83%) ethambutol, and 68 (69%) pyrazinamide.

The most common presenting symptoms at the onset of AKI were skin rash (21%) and gastro-intestinal disturbance (17%), followed by fever (6%) and arthralgia (4%) (Table 1). The most common laboratory findings were hypoalbuminemia, increased eosinophil count (>0.5×10^9^/L), and anemia (hemoglobin < 100 g/L) (Table 2). Urinalysis showed proteinuria in 20%, sterile leukocyturia in 17%, and hematuria in 5%. Aside from elevated serum creatinine level, serum uric acid level was also elevated during AKI compared to baseline (*p* < 0.001) (Table 2).

**Table 2:** Laboratory data of patients who did and did not recover from acute kidney injury (AKI)

	**No. of patients with data**	**Overall (n = 99)**	**AKI-recovered (n = 71)**	**AKI-unrecovered (n = 26)**	**Loss of follow-up (n = 2)**
Baseline					
Creatinine (μmol/L)	99	88.4 [55.2-132.6]	88.4 [70.7-132.6]	88.4 [61. 9–150.3]	62 [53–71]
Uric Acid (μmol/L)	82	374.7 [285.5-440.2]	374.7 [267.7-434.2]	377.7 [339.0-493.7]	485 [232–738]
Onset of AKI					
Creatinine (μmol/L)	99	123.8 [97.2-246.8]	123.8 [97.2-159.1]	132.6 [106.1-238.7]	122 [84–159]
Blood urea nitrogen (mmol/L)	76	8.4 [6.0-15.4]	7.5 [5.6-13.3]	14.5 [7.9-20.7]*	NA
Uric Acid (mmol/L)	83	529.4 [386.6-678.1]	535.32 [386.6-695.9]	499.6 [350.9-565.1]	518 [440–595]
Hemoglobin < 100 (g/L)	84	22 (26)	15 (25)	7 (33)	0 (0)
Eosinophil >0.5 (10^9^/L)	73	21 (29)	14 (25)	7 (44)	0 (0)
White blood cell >10 (10^9^/L)	85	15 (18)	11 (18)	4 (19)	0 (0)
Platelet < 100 (10^9^/L)	85	9 (11)	9 (15)	0 (0)**	0 (0)
Hepatitis^#^	97	4 (4)	3 (4)	1 (4)	0 (0)
Jaundice^§^	81	3 (4)	2 (3)	1 (6)	0 (0)
Hypoalbuminemia	94	39 (41)	24 (36)	14 (54)	1 (50)
Hematuria	35	5 (5)	2 (7)	3 (38)*	0 (0)
Proteinuria	35	20 (20)	13 (48)	7 (88)*	0 (0)
Sterile leukocyturia	35	17 (17)	13 (48)	4 (50)	0 (0)

Note: Data are either median [inter-quartile range] or number (%) unless otherwise stated.

*Significantly different (*p* < 0.05) between the AKI-recovered and –unrecovered groups.

**p = 0.064.

^**#**^Hepatitis was defined as increased serum alanine aminotransferase >3 times the upper limit of normal (ULN) in symptomatic, or >5 times the ULN in asymptomatic patients.

^§^Jaundice was defined as serum total bilirubin level >51.3 μmol/L.

### Modifications of anti-TB treatment during AKI

After the onset of AKI, rifampin was discontinued in 34 (34%) patients (Table 1). Among them, re-challenge was performed in 21 (62%), with six (18%) developing a second episode of AKI (Table 1). Of the remaining 13 patients who did not undergo rifampin re-challenge, six were treated with regimens not including rifampin, while five had clinical observation only (without anti-TB medication). The remaining two were lost to follow-up. Overall, rifampin was successfully re-introduced or continued without interruption in 87% of the 71 AKI-recovered patients and in 77% of the 26 AKI-unrecovered patients (Figure 1).

Pyrazinamide was discontinued in 50 (51%) patients after the onset of AKI. Anti-TB drugs were interrupted in 30 (30%), including seven (7%) who failed to complete the treatment (four stopped due to culture negativity, one died, and two were lost to follow-up). In the remaining 23 patients, the median duration of treatment interruption was 14 days (IQR, 7–28 days).

### Outcome and prognostic factors of AKI

Within a follow-up period of 180 days since the onset of AKI, two patients were lost. Twenty-six (27%) did not recover from AKI (AKI-unrecovered group), including six (6%) (median age, 68.5 years [IQR 59–81.5 years]) who required long-term renal replacement therapy. The median baseline creatinine level of the six patients was 221 μmol/L (IQR, 141.4-318.2 μmol/L), significantly higher than those of the other 93 patients (*p* = 0.002). Five of the six patients had hypoalbuminemia.

Among the 71 patients who recovered from AKI (AKI-recovered group), 64 (90%) recovered within 100 days. Serum BUN level (*p* = 0.005) and the prevalence of hematuria (*p* = 0.033) and proteinuria (*p* = 0.048) at the onset of AKI were significantly higher in the AKI-unrecovered group, whereas thrombocytopenia was less common (*p* = 0.064) (Tables 1 and 2). The AKI recovery rate was not different among the different AKIN stages (*p* = 0.061) (Table 1).

**Figure 3:**
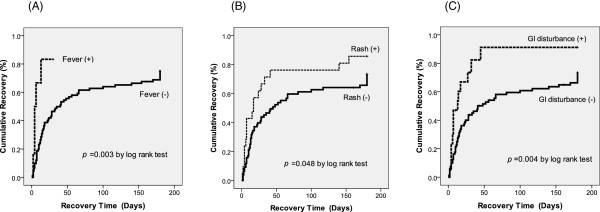
**Kaplan-Meier curves for time to recovery from acute kidney injury among patients with or without (A) fever, (B) rash, and (C) gastro-intestinal (GI) disturbance.** Sub-group difference was compared by log-rank test.

In the Kaplan-Meier analysis, fever (HR 3.65 [1.43-9.37]), gastro-intestinal disturbance (HR 2.32 [1.27-4.27]), and thrombocytopenia (HR 2.20 [1.08-4.50]) at the onset of AKI were significant predictors of renal recovery, whereas skin rash (HR 1.69 [0.99-2.90]) and arthralgia (HR 2.78 [0.99-7.78]) had borderline significance (Figure 3). Multivariate Cox proportional hazard regression analysis including all of these five variables revealed that the VIFs of all variables were < 3. The independent predictors of renal recovery were fever, gastro-intestinal disturbance, and skin rash (Table 3).

**Table 3:** Predictive factors of recovery from acute kidney injury (AKI), by multivariate Cox proportional hazard regression analysis

**Variables**	**Median days for AKI recovery**	***p *** **value**	**HR**	**95% CI**
Fever at onset of AKI: yes vs. no	4 vs. 40	0.013	3.43	1.29-9.12
Rash at onset of AKI: yes vs. no	17 vs. 45	0.044	1.79	1.02-3.14
GI disturbance at onset of AKI: yes vs. no	13 vs. 41	0.023	2.07	1.11-3.89

Sensitivity analysis focusing on the sub-population without CKD revealed that fever at AKI onset remained a significant predictor of renal recovery (*p* = 0.002; HR 11.99 [2.43-59.19]), whereas thrombocytopenia (*p* = 0.051; HR 2.40 [1.00-5.78]) and gastro-intestinal disturbance (*p* = 0.091; HR 1.94 [0.90-4.17]) had borderline significance.

## Discussion

Acute kidney injury is a rare complication in patients on anti-TB therapy. Several reports, summarized in four review articles [4,11-13], address this rare event and most reveal rifampicin as the most common responsible drug. By retrospective analysis of 2322 TB patients, this study has several important findings. First, the incidence of AKI in patients on anti-TB therapy is much higher in Taiwan (7.1%) than in previous reports (0.05%) [11], with a recovery rate of 73% (>80% in literature) [4,11-13]. Second, rifampin is successfully re-introduced or continued without interruption in 87% of TB patients with AKI recovered. Third, factors predicting recovery from AKI are clinical symptoms at the onset of AKI, including fever, skin rash, and GI disturbance, but not AKI stage, age, and co-morbidities.

The definition of rifampin-induced renal impairment varies in previous studies [4,11-13,21]. The exact incidence rate is unknown. Only the review article from Romania reports that 0.05% of patients receiving rifampin (mean age, 45 years) develop acute renal failure, defined as elevated serum creatinine >44.2 μmol/L or >20% of baseline in two weeks [11]. Using the criteria established by the AKIN [16], AKI during anti-TB treatment in the current study is not uncommon (7.1%), probably reflecting the old age (mean age, 65.9 years) and high prevalence of systemic co-morbidity, such as DM and CKD, that can predispose to more kidney damage [14].

The findings that 60% of patients are older than 65 years and 80% have positive mycobacterial culture are similar to the country-wide epidemiologic data reported by the Taiwan Center of Disease Control (TCDC) (age >65 years, 52%; culture-positive rate, 80%), implying that the study subjects here are representative of the whole TB population in Taiwan [3]. With the global trend of aging, determining the local incidence rate of AKI is necessary to improve the quality of TB care and to determine the frequency and duration of monitoring.

The mechanism of rifampin-induced AKI is not well established. Several studies suggest that it is either a type II or type III hypersensitivity reaction induced by rifampin antigens in which anti-rifampin antibodies form immune complexes that are deposited in renal vessels, the glomerular endothelium, and the interstitial area [12]. These reactions cause two different pathologic changes in the kidneys. The deposition of immune complexes in the vessels causes vascular constriction and tubular ischemia, leading to acute tubular necrosis, whereas the deposition of immune complexes in the interstitial area leads to acute interstitial nephritis [22]. Renal biopsies performed in several studies with a total of 106 patients reveal that the most common pathologies are acute interstitial nephritis (54%) and acute tubular necrosis (38%) [4,11-13]. The immune reaction is indirect proof by the Romania study of a positive correlation between the duration of anuria and serum gamma-globulin level [11].

In previous studies, more than 80% of patients recover from AKI within 120 days [4,11-13]. The recovery rate in the present study (73%) is slightly lower, probably due to the older age and the presence of underlying co-morbidities. Because AKIN stage includes mild renal impairment, some patients who improve their renal function but still fulfill the stage I criteria of AKI may be classified as “unrecovered”. One report reveals that age may predict delayed renal function recovery in patients with drug-induced acute interstitial nephritis [15]. However, the present study has different findings. The recovery time of AKI-recovered patients is similar to those of previous reports, with 90% recovery within 100 days [4,11-13]. Thus, close monitoring and avoidance of further kidney injury for three-to-four months after the onset of AKI during anti-TB treatment are necessary.

The prognostic factors of AKI during anti-TB treatment are rarely investigated. Only the duration of anuria and leukocytosis have been associated with renal recovery [11]. The current study lacks data on the duration of anuria and few patients (n = 33) underwent urinalysis. After including clinical symptoms, demographic data, and laboratory results into the statistical model, the multivariate Cox regression analysis reveals that the presence of fever, rash, and GI disturbance at the onset of AKI are associated with better renal recovery. Because fever and skin rash are common manifestation of acute interstitial nephritis [23], the underlying pathophysiology of AKI in patients with these two symptoms is more likely to be acute interstitial nephritis. Since acute interstitial nephritis has better prognosis than acute tubular necrosis, these patients also have better renal recovery [24,25]. For patients with GI disturbance, AKI may be partly due to dehydration and hypo-perfusion. With careful fluid management, renal impairment may be quickly overcome.

More than 50% of the AKI events occurred within two months of anti-TB treatment, indicating that an acute phase reaction may be contributory. The findings also suggest that patients with CKD and hypoalbuminemia maybe more vulnerable to severe and permanent renal damage. After AKI develops, more physicians decide to discontinue pyrazinamide, rather than rifampin, implying that they do not know which of the first-line anti-TB drugs is the most common offending drug for AKI. Continuous medical education on the correct regimen modification is necessary to prevent further renal damage in TB patients with AKI.

In this study, the diagnosis of AKI is not confirmed because renal biopsy was not performed. However, the results of previous studies suggest that even without histology studies, the diagnosis of rifampin-induced AKI can be made based on the typical time course and by excluding other etiologies [11]. In the present study, the medical records were reviewed extensively to exclude other possible causes of AKI like sepsis, hypotension, or use of other nephrotoxic medication. Seven patients had a second AKI episode after rifampin re-challenge, further confirming that rifampin may be the leading cause of AKI.

Re-treatment or re-exposure to rifampin causes repeat antigen exposure, which can lead to a high antibody surge and subsequent severe immune response [11,26]. This theory is supported by the finding that a high percentage of patients with rifampin-induced AKI are re-treatment cases [4,11-13]. However, the findings here are different from previous observations and show that only 11% of AKI patients are re-treatment cases. Rifampin has been successfully re-introduced in 71%. The possible explanation is drug desensitization [26]. Although the rifampicin desensitization protocol varies, success rates (80-82%) of re-introducing rifampin are high in some studies [27-30]. Further large-scale studies are needed to address whether re-exposure to rifampin is an independent risk factor of developing AKI, and to determine the method of rifampin re-introduction.

The present study has some limitations. First, there is no strong evidence to confirm rifampin as the cause of AKI due to the lack of pathology results. Only seven patients had a second AKI episode after re-challenge rifampin. However, this may not be a serious problem because possible causes other than anti-TB medication have been excluded and AKI due to first-line anti-TB drugs other than rifampin is rarely reported [5,6]. Second, in this retrospective study, there is no standard protocol of laboratory follow-up for every TB patient during anti-TB treatment. Follow-up depends on the primary care physicians. Patients who did not have any symptoms or signs suggestive for AKI usually had no follow-up data on renal function. Therefore, risk factors of AKI during anti-TB treatment were not identified. Furthermore, asymptomatic patients with AKI may be missed, resulting in lower incidence and recovery rates of AKI. Third, although some characteristics of the study subjects are similar as those of the general TB population in Taiwan, the results here may not be applicable to all TB patients because this is a retrospective study conducted in a medical center.

## Conclusions

Anti-tuberculosis drug-induced acute kidney injury is not rare in an aging population. It usually develops within two months of treatment and resolves within three months after onset. Although about 27% of patients with AKI will have permanent renal impairment, those who present with fever, rash, and GI disturbance at the onset of AKI have better renal recovery. Of the 73% of patients who had recovery of renal function, 87% successfully continued rifampicin or had rifampicin re-introduced.

### Ethical approval

The study was approved by the Institutional Review Board of National Taiwan University Hospital (NTUH_IRB: 9561707008).

### Summary

Tuberculosis is a global disease affecting one-third of the world’s population. Acute kidney injury is not uncommon in patients receiving anti-tuberculosis drugs. Identifying risk and prognostic factors of acute kidney injury is important in the management of tuberculosis.

## Competing interests

The authors declare that they have no competing interests.

## Authors’ contributions

CHC, YFC, VCW, JYW: study concept and design, analysis of data, and drafting of manuscript. CCS, CHL: statistical analysis and interpretation of data. CHC, JYW: interpretation of data and review of manuscript. LNL, CJY: review of manuscript. All of the authors have seen and approved the final version of the manuscript.

## Pre-publication history

The pre-publication history for this paper can be accessed here:

http://www.biomedcentral.com/1471-2334/14/23/prepub
